# Biochemical principles of SMAD signaling across the animal kingdom

**DOI:** 10.1042/BCJ20250135

**Published:** 2026-07-16

**Authors:** Razeen Shaikh, Caitlin Frank, Nissa J. Larson, Hung-Yuan Chen, Anuj Girish Pradhan, Kailee Mendiola, Donny Hanjaya-Putra, David M. Umulis, Jeremiah Zartman, Gregory T. Reeves, Linlin Li

**Affiliations:** 1 Artie McFerrin Department of Chemical Engineering, Texas A&M University, College Station, Texas, TX, U.S.A.; 2 Bioengineering Graduate Program, University of Notre Dame, Notre Dame, IN, U.S.A.; 3 Chemical and Biomolecular Engineering, University of Notre Dame, Notre Dame, IN, U.S.A.; 4 Weldon School of Biomedical Engineering, Purdue University, West Lafayette & Indianapolis, IN, U.S.A.; 5 Aerospace and Mechanical Engineering, University of Notre Dame, Notre Dame, IN, U.S.A.; 6 Biological Sciences, University of Notre Dame, Notre Dame, IN, U.S.A.; 7 Interdisciplinary Graduate Program in Genetics and Genomics, Texas A&M University, College Station, TX 77843, U.S.A.

**Keywords:** Drosophila, BMP Pathway, pluripotent stem cell, systems biology, Zebrafish

## Abstract

The bone-morphogenetic protein (BMP)-SMAD signal transduction pathway regulates fundamental cellular processes such as fate specification, tissue patterning, and stem cell homeostasis across metazoans and has a conserved signaling architecture. However, the quantitative dynamics of SMAD signaling and regulatory strategies governing pathway activity show a wide range of variation across developmental and stem-cell systems. In the present review, we summarize insights from six major biological contexts—*Drosophila* embryo, germline stem cells, and the larval and pupal wing discs; the *Danio rerio* (zebrafish) embryo; and human pluripotent stem cells—to compare how BMP signals are measured, manipulated, modeled, and integrated. We begin by outlining the canonical BMP signaling pathway and the mechanisms of BMP gradient formation across developmental systems, highlighting how conserved pathway components contribute to the formation of system-specific spatial profiles. We then summarize intracellular Smad dynamics and how endogenous pathway dynamics are measured through quantitative imaging of the phosphorylated Smad. Next, we examine how BMP signaling is interpreted through tiered transcriptional responses of downstream target genes. Next, we summarize the mechanistic and computational models of integrated gradient formation, signal transduction, and gene regulation across the presented systems. These insights reveal unifying design principles and performance objectives that govern BMP-SMAD signaling across species and cell types and frame open questions for future cross-species and translational studies.

## Introduction

**Figure 1:**
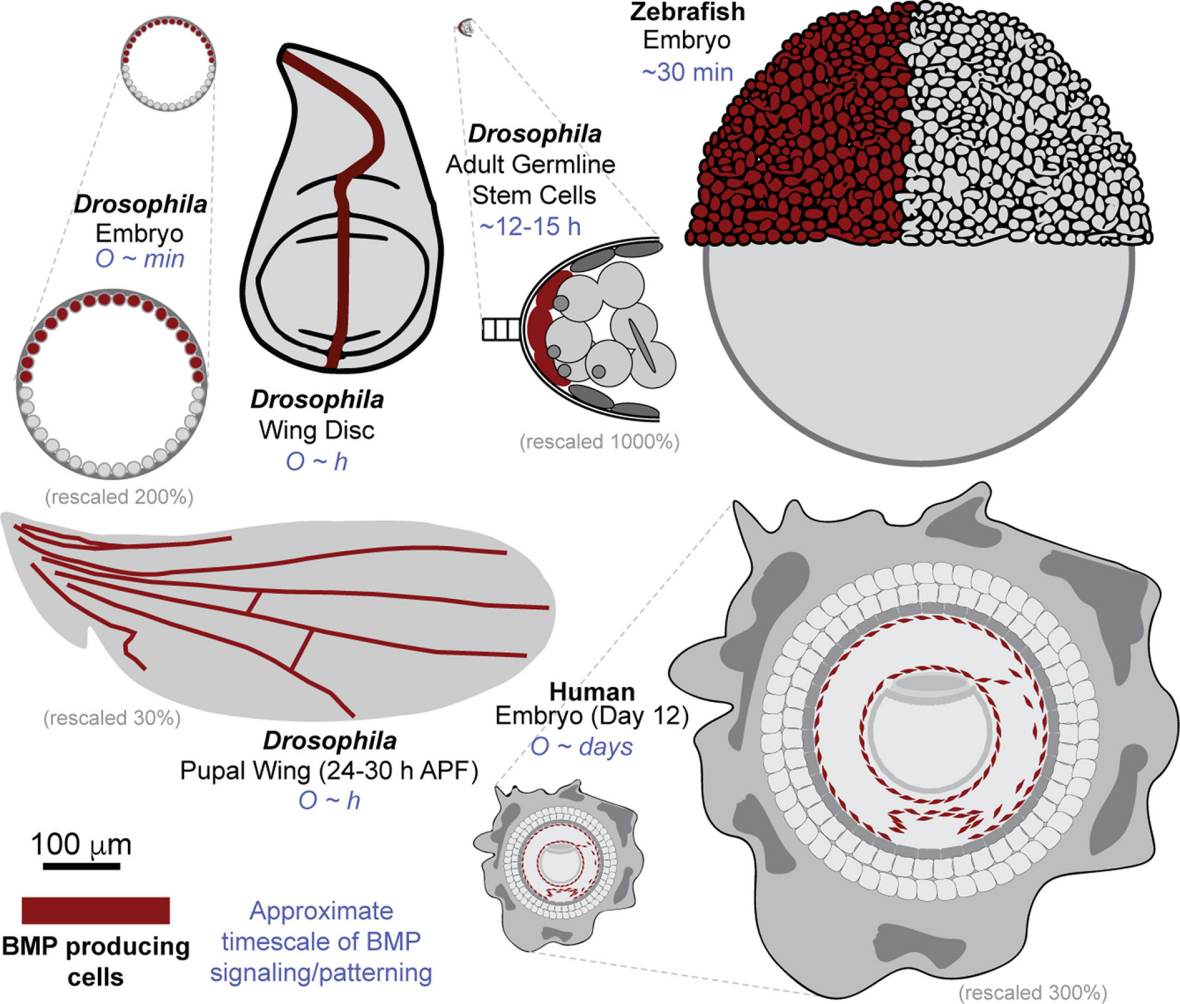
Spatial patterning of BMP ligands is a common theme across development from *Drosophila* to human systems The cells that produce BMP ligands are in parenthesis for each model system and illustrated in maroon. Examples include patterning in the *Drosophila* embryo (Dorsal half), larval (a stripe of cells situated at the boundary between the anterior and posterior compartments) and pupal wing anlage (wing veins), and the adult female germline stem cells (cap cells); Zebrafish embryo (ventral) and the human embryo (extraembryonic mesoderm). The figure highlights the diversity in length and timescales across developmental contexts.

The transforming growth factor β (TGF-β) superfamily of growth factors regulates homeostasis, differentiation, and regeneration and is highly conserved throughout the animal kingdom. Ligands from the TGF-β superfamily include TGF-β isoforms, bone-morphogenetic proteins (BMPs), growth and differentiation factors (e.g., activin and nodal). We aim to review the quantitative advances in understanding the BMP-Smad pathway by primarily focusing on the work performed in six model systems: the *Drosophila* embryo, germline stem cells, and larval and pupal wing disks, the zebrafish embryo, and human pluripotent stem cells (hPSCs) (Figure 1).

**Figure 2:**
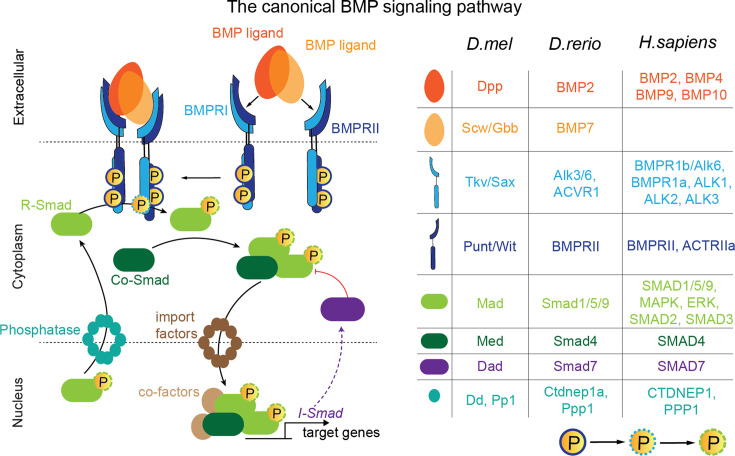
The core conserved network of BMP signaling This figure illustrates the canonical BMP signal transduction pathway topology (**A**) and the core molecular players across three species: *Drosophila melanogaster, Danio rerio*, and *Homo sapiens* (**B**). (A) The BMP ligands bind to their cognate transmembrane receptors forming a heterodimer-tetramer complex. This enables the phosphate group on Type II receptor to phosphorylate the Type I receptor and finally phosphorylate the intracellular R-Smad forming ‘pSmad’ (the outline colors on the phosphate group indicate its association with either receptor or Smad). pSmad undergoes several interactions with Co-Smad to form dimeric and trimeric complexes. The trimeric Smad complex recruits several co-factors which enable DNA binding and regulation of target genes - including I-Smad forming a negative feedback loop. (B) The table lists core members of the BMP/Smad pathway and their orthologs in *Drosophila melanogaster (D. mel), Danio rerio (D. rerio)*, and *Homo sapiens (H. sapiens).*

The BMP pathway regulates a broad range of developmental processes across model systems (Figure 1). In *Drosophila*, it patterns the dorsal/ventral axis in the embryo, defines the anterior-posterior boundary in the larval wing disc, contributes to vein differentiation in the pupal wing, and regulates maintenance and differentiation in germline stem cells. Likewise, in its vertebrate counterparts (zebrafish and hPSCs), this pathway governs embryonic axis formation, lineage specification, and cell-fate decisions [1]. The core members of the BMP pathway include the BMP ligands, their cognate Type-I and Type-II receptors, and the intracellular receptor-regulated (R-SMAD) and common mediator SMADs (Co-SMADs). In the canonical SMAD pathway, the extracellular BMP ligands (BMP2, BMP4, and BMP7) dimerize and bind to the Type-I/Type-II transmembrane serine/threonine kinase receptors, which trigger intracellular phosphorylation of R-SMAD. Phosphorylated R-SMAD then interacts with Co-SMAD to form a trimeric transcription factor that regulates the expression of several downstream target genes (Figure 2).

The structure and function of the TGF-β/BMP receptors and ligands are highly conserved; as such, human BMP ligands can replace their orthologs in *Drosophila* [2,3]. The homologs of the BMP ligands (BMP2, BMP4), Type I receptors (ALK2, ALK3, and ALK6), Type II receptors (BMPR2 and ACVR2), R-Smads (Smad 1/5/9), and Co-Smad (Smad4) are prevalent across the vertebrate systems, including zebrafish and humans (Table 1 and Figure 2). The orthologs of BMP components in *Drosophila* include decapentaplegic (Dpp; BMP2/4 ortholog) and glass bottom boat (Scw/Gbb; BMP5/6/7/8 ortholog) ligands, the Type-I receptors thickveins (Tkv) and saxophone (Sax), the Type-II receptors Punt (Put) and wishful thinking (Wit), and the intracellular Smads, Mothers against Dpp (Mad; Smad1/5/9 ortholog) and Medea (Med; Smad4 ortholog) (Table 1 and Figure 2). The signaling pathway recruits several extra- and intracellular mechanisms to distinctly regulate its downstream target genes in a tissue-specific, context-dependent manner across length and time scales ranging from a few microns in the adult *Drosophila* germline stem cells to several millimeters in the zebrafish embryo and from minutes in the *Drosophila* embryo to days in the pupal wing (Table 1). However, we note that the term ‘BMP gradient formation and time scale’ for the *Drosophila* germline stem cell (GSC) niche, because of its short length scale and lack of sufficient quantitative information, needs further justification. The majority of Dpp available to the GSC niche is produced by the cap cells. Further, Wilcockson and Ashe (2019) used a Dpp-mCherry line and quantified a higher Dpp concentration in the anterior germarium. Additionally, the time scale over which cytoneme projections from the GSCs capture Dpp from the cap cells is on the order of minutes. Based on this evidence, we can conclude that there is an extracellular gradient of Dpp in the GSC niche, and Dpp released from the cap cells is available to the receptors on the GSCs on the order of minutes.

**Table 1:** Dynamics and functional outputs of SMAD network across biological systems

BMP pathway component and properties	*Drosophila*: 1. Blastoderm Embryo	*Drosophila:* 2. Larval Wing Disc	*Drosophila*: 3. Pupal Wing	*Drosophila*: 4. Germline stem cells	*Zebrafish:* 5. Embryo	*Human:* 6. Endothelial cells differentiated from hPSCs
**Downstream target/ differentiation marker**	Type I target: *zen, Race*, and *hnt* Type II target: *rho, tup*, and *ush* Type III target: *pnr* [4]	*Brinker (brk), optomotor-blind (omb), daughters against dpp (dad)*, and *spalt major (sal)* [5,6]	*Dad* [5,6] and *Crossveinless-2 (CV-2)* (*BMP-responsive extracellular regulator)* [7,8] *EGFR/MAPK outputs (vein differentiation): Rhomboid (rho), Star (s), and argos (aos) (indirect)* [9,10], *and blistered/Srf (bs) (repressed)* [11]	*dad, Rfx, and futsch, bag of marbles (bam)* [12]	*highest threshold: sizzled (szl) and tp63* *Intermediate threshold: foxi1 and gata2a* *Low threshold: bambia* [13,14]	ID1, ID2, ID3 SMAD6/7 [15]
**BMP Gradient Formation Time Scale**	Tens of minutes after nuclear cycle 13 [16,17]	<4 h [18]	Hours (∼18–30 h APF) [19,20]	Minutes (rapidly) [12,21]	Hours [22]	Not applicable^*^ (uniform BMP signaling)
**Gene expression time scale**	∼30 min [23]	Not directly measured/TBD	Hours (Overlaps with gradient formation and sharpens at later pupal stages) [19]	Hours (not directly measured) [8]	Minutes to hours [13,14]	≤24 h (endpoint measured; onset not resolved) [24]
**Gradient length scale**	5–6 cell diameters (35 μm) [23]	20–40 cell diameters (100 μm) [22]	Short-range: vein restricted cell-scale, ∼1–3 cell diameters (∼5–15 μm) [10,20] Long-range: Transport to crossveins (∼50–100 μm) [15,21]	1–2 cell diameters (5 μm) [12,21,22]	25+ cell diameters (∼700 μm) [22]	Not applicable^*^ (uniform BMP signaling)

* For *in vitro* human pluripotent stem cell systems, BMP is applied uniformly and does not form a spatial gradient; hence, reported time scales reflect transcriptional endpoints rather than resolved signaling kinetics.

BMPs are tightly controlled during development and in homeostasis, since dysregulation can result in developmental defects or disease. Regulation commonly occurs through inhibitory Smads (I-Smads), phosphatases (PPases), and E3 ubiquitin ligases, which are also conserved across these six model systems (Figure 2). The I-Smads (Smad6/7; *Drosophila* ortholog daughters against Dpp, Dad) introduce negative feedback to the pathway topology as they are transcriptionally up-regulated by the BMP signal transduction and terminate BMP signaling by interacting with the activated receptors or R-Smads [25]. Phosphatases regulate the BMP pathway through dephosphorylation of receptors or other downstream components to terminate the signaling cascade. Key phosphatases, such as CTDNEP1 (C-terminal domain nuclear envelope phosphatase 1 (Dullard (Dd)), are also conserved across species and are suspected to regulate the pathway through the dephosphorylation of Type I receptors or R-SMADs; however, it is worth noting that these phosphatases are not specific to the BMP pathway and also dephosphorylate other components in the Wnt pathway [26–28]. Likewise, E3 ubiquitin ligases regulate this pathway by promoting the proteolysis of Smads [26,29]. This includes the SMURF family of E3 ubiquitin ligases, which are major negative regulators of the BMP pathway in human induced pluripotent stem cells (hiPSCs), zebrafish embryos, and *Drosophila* [29–31]. The complexity of these interactions underscores the importance of continued research into the mechanisms of pSmad regulation to fully elucidate the intricacies of TGF-β/BMP signaling.

The mechanism through which extracellular BMP gradients are formed is highly context-dependent, including combinatorial signal perception and regulation through BMP inhibitors, enabling adaptation to specific developmental contexts, ranging from short-range signaling in *Drosophila* female germline stem cells to long-range signaling in the *Drosophila* wing disc [32–34]. Despite the diversity of extracellular cues that activate the BMP pathway, the intracellular SMAD module that interprets these signals is remarkably conserved, functioning as a dynamic decoder that transforms spatial gradients and temporal fluctuations into precise transcriptional outputs. There is growing evidence from both experimental and computational studies that SMAD signaling is a dynamic process, including dynamic signal processing [31,32,35], which provides a more complex function that cannot be captured through typically quantitative approaches that measure BMP concentration. The Smads dynamically shuttle between the nucleus and the cytoplasm and, upon BMP stimulation, localize to the nucleus [36]. Analogous to BMP signaling, Schmierer et al. demonstrated that in the TGF-β pathway, rapid and continuous shuttling of Smad proteins between the cytoplasm and nucleus enables cells to effectively integrate signaling over time, filters noise, and allows cells to detect transient TGF-β inputs [37]. Findings in human embryonic stem cells (hESCs) demonstrate that rapid changes in BMP concentration are key drivers of self-organized patterning through their effects on pSMAD dynamics and downstream WNT/NODAL activation [38]. These insights indicate that the robustness and versatility of BMP/TGF-β signaling arise not only from classical inhibitory controls but also from the dynamic signal-processing capabilities, which allow conserved intracellular machinery to generate diverse developmental outcomes as a modifiable signaling module.

Here, we summarize the recent quantitative understanding of the mechanisms that confer diverse modalities in the TGF-β/BMP pathway tuned for dynamic performance across model systems. These include the vertebrate model systems, zebrafish and mammals, and the invertebrate *Drosophila* systems, germline stem cells, embryo, wing disc, and the adult wing, each of which functions under different time and space constraints (Table 1). First, we review the mechanism of gradient formation and highlight the similarities and differences in gradient diffusion lengths, signaling schemes, and underlying feedback loops across the six model systems (in the ‘Mechanisms of extracellular BMP distribution and receptor-level regulation’ section). Second, we summarized the intracellular Smad dynamics and highlighted that pSmad has conventionally been used as a proxy for extracellular TGF-β/BMP gradients and evaluated the quantitative live imaging approaches to record endogenous expression levels (in the ‘Intracellular Smad dynamics and signal processing’ section). Third, we examine how BMP activity is interpreted through a tiered transcriptional response of downstream target genes (in the ‘Interpretation of BMP signals via transcriptional regulation’ section). Fourth, we perform a meta-analysis of the mechanistic and computational models of the pathway across the chosen model systems (in the ‘Computational modeling approaches that drive mechanistic and cross-species insights’ section). Fifth, we synthesize how gradient formation, signal interpretation, and modeling converge to reveal unified design principles and performance objectives (POs) for the BMP-Smad pathway (in the ‘Pathway level performance objectives in system-specific contexts’ section). Finally, we discuss performance objectives of BMP signaling across diverse biological contexts and propose how experimental data and computational models can be integrated to better understand the versatile and dynamic regulation of this pathway across diverse biological contexts (in the ‘Discussion and open questions’ section).

## Mechanisms of extracellular BMP distribution and receptor-level regulation

Morphogens provide positional information to cells by spatially varying their concentration across tissues. In the BMP pathway, these gradients arise through a combination of ligand diffusion, extracellular modulation, receptor interactions, and feedback control. Overall, the core signaling machinery of the BMP pathway is highly conserved across the described size systems; however, subtle variations in shuttling dynamics and regulatory proteins occur to account for the scale differences in tissue architecture. A summary of the six systems of interest is provided below.

### Drosophila blastoderm embryo

**Figure 3:**
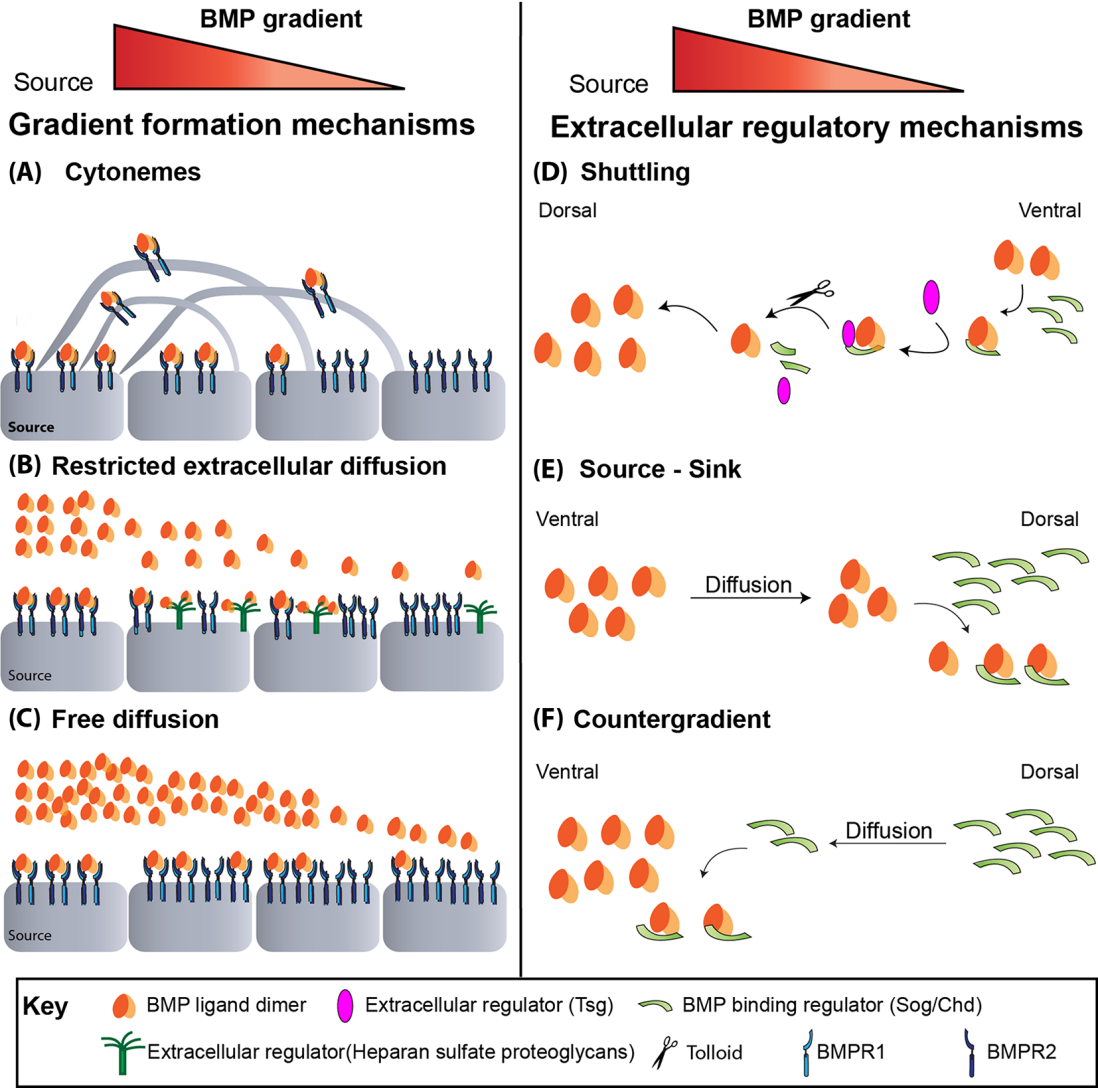
Summary of proposed mechanisms of gradient formation across systems The concentration of BMP decreases from left to right, as shown by the top figure. (**A**) Gradient formation by cytonemes: Cytonemes are thin, cellular projections that can emanate from the cell towards the morphogen source. (**B**) Gradient formation by restricted extracellular diffusion: Extracellular proteins in the matrix regulate and restrict free diffusion. (**C**) Gradient formation by free diffusion: The BMP ligand diffuses from regions of high concentration to low concentration. (**D**) Shuttling mechanism, as employed in *Drosophila* embryo and pupal wing; BMP regulator, Sog concentrates BMP towards the dorsal midline. (**E**) Source-sink mechanism, as employed in zebrafish, where the BMP regulator, Chordin, acts as a dorsal sink for the freely diffusing BMP. (**F**) A countergradient, as proposed in zebrafish, where the BMP binding regulator, Chordin, diffuses to regulate BMP activity.

In *Drosophila*, the mechanism behind Dpp gradient formation changes during different stages of development. In early *Drosophila* embryogenesis, approximately 2 h after fertilization, BMP begins as a broad and weak signal and, within 20–30 min, refines into a sharp, localized dorsal pMad domain [7,16,39]. This refinement is initiated by extracellular shuttling, in which short gastrulation (Sog) and twisted gastrulation (Tsg) bind BMP ligands and facilitate their dorsal transport, while Tld-mediated proteolytic cleavage releases active ligands near the dorsal midline. This initial broad BMP activity profile is then further driven by an intracellular positive feedback loop that enhances BMP binding at the cell surface, which allows the dorsal midline cells to outcompete lateral cells for BMP ligands and effectively sharpen the signaling boundary [40]. BMP ligands Dpp and Scw are expressed broadly [41]; however, their spatial activity is modulated by extracellular inhibitors (Sog and Tsg, which bind to Dpp/Scw and prevent receptor activation in lateral regions. The Dpp/Scw heterodimers, and not their respective homodimers, predominantly activate the pathway due to their higher affinity for Sog, Tsg, and the Sog/Tsg complex, inferred through co-immunoprecipitation assays, and they also render robustness to gene dosages [42]. These ligand-inhibitor complexes diffuse dorsally, where the metalloprotease Tolloid (Tld) cleaves Sog, releasing active Dpp/Scw ligands near the dorsal midline. This facilitated diffusion mechanism, more commonly known as ‘shuttling,’ concentrates BMP ligands dorsally [42]. Early modeling efforts demonstrated that canonical pMad gradient formation in the early embryo requires the presence of the metalloprotease Tld and BMP antagonists (Sog and Tsg) through shuttling and Tld-mediated cleavage [23,43–45]. Despite the success of this model in recapitulating *in vivo* behavior, diffusion-degradation models have demonstrated that ligand diffusion alone cannot account for the sharp signaling domain or that BMP initially accumulates at the dorsal midline before gradually intensifying and broadening both in amplitude and spatial extent [7]. To improve on this model, Umulis et al. advocated that incorporating feedback regulation and receptor-mediated degradation with shuttling models (Figure 3D),was necessary to achieve the rapid gradient sharpening and spatial bistability seen in the system [40]. Specifically, the concepts of spatial bistability and intracellular positive feedback were adopted from Wang and Ferguson, whose experimental results showed the dependence of spatial patterning upon this positive feedback [46]. In a follow-up study by Umulis et al., 3D modeling of the *Drosophila* embryo further showed the consistency between the suggested feedback regulation mechanism and the experimental data [47].

Additionally, the *Drosophila* embryo also exhibits scale invariance. Dorsal surface patterning by Dpp/BMP scales between closely related species and between individuals within a species; specifically, the ratio of pMad pattern width to embryo length is constant between *Drosophila melanogaster*, the larger *Drosophila virilis*, and the smaller *Drosophila busckii*, as well as among individual embryos of differing sizes within each species [47].

### Drosophila larval wing disc

During larval wing disc development, Dpp is expressed as a stripe across the middle of the imaginal disc, which forms a long-range gradient along the anterioposterior axis that patterns the longitudinal veins (LVs) [48]. The major competing hypotheses regarding the long-range Dpp gradient formation in *Drosophila* wing discs are through receptor-mediated transcytosis (RMT), active transport mechanisms involving cytonemes, and restricted extracellular diffusion (including hindered diffusion) (Figure 3A–C) [49,50]. According to the RMT hypothesis, long-range distribution and transport of Dpp occur through repeated receptor-mediated uptake and secretion.

RMT was a leading hypothesis in the Dpp gradient formation in the wing disc; however, more recent work has suggested that RMT cannot account for rapid Dpp diffusion and may have a limited role in Dpp transport [50]. Cytoneme-mediated active transport is another proposed mechanism of Dpp movement and involves cellular projections that directly connect the ligand-receiving to ligand-producing cells (Figure 3A). The role of cytonemes in Dpp gradient formation in wing discs has not been conclusively established. More recent work has shown direct imaging of cytonemes in the adjacent air sac primordium [51]. Recent studies support restricted extracellular diffusion as the major mechanism behind Dpp gradient formation in the larval wing disc (Figure 3B). During restricted extracellular diffusion, extracellular matrix (ECM) proteins—glypicans—(e.g., glycosylphosphatidylinositol (GPI)-anchored heparan sulfate proteoglycans) act as ligand sinks upon interacting with Dpp to facilitate Dpp cellular endocytosis [49,52]. One study investigating minimal requirements for morphogen gradient formation successfully recapitulated Dpp gradient formation behavior when using an inert synthetic morphogen (GFP) that is unable to diffuse via specialized transport mechanisms (e.g., planar transcytosis) [52]. Kinetic modeling of GFP’s interaction with signaling receptor dimers and GPI-anchored non-signaling receptors demonstrated that free and GPI-anchored assisted diffusion were responsible for the Dpp-like gradient, indicating that diffusion mechanisms and not transcytosis are responsible for the Dpp gradient formation [52]. Another study directly investigating RMT and restricted extracellular diffusion in Dpp gradient formation further supported this argument by demonstrating that long-range distribution of Dpp occurs through restricted extracellular diffusion when utilizing computational modeling and gain-of-function and loss-of-function Tkv receptor mutant clones [49].

To better demonstrate this phenomenon, other gradient formation models attempt to combine extracellular diffusion and receptor/matrix interactions to explain the mechanism of the Dpp gradient formation through the wing disc via temporal regulation of gene expression. In these models, Dpp levels are highest near the anterior-posterior axis and reduced laterally due to the gradient formation [53,54]. These models further explore the hypothetical scenario in which Dpp levels are made uniform across the tissue [49]. Under this assumption, lateral cells, which normally receive lower Dpp, are predicted to experience increased proliferation due to their lower response threshold, whereas medial cells, which are normally exposed to high Dpp levels, will experience less proliferation. However, these models fail to recapitulate *in vivo* behavior where proliferation is uniform across the wing disc, despite the nonuniform Dpp gradient [48,49]. The Dpp gradient in the *Drosophila* wing disc also exhibits scale invariance via dynamic scaling, as characterized in Ben-Zvi and Barkai (2010), who proposed the expansion-repression model, where a secreted inhibitor Pentagone (Pent) acts as a rapidly diffusing ‘expander’ molecule of the Dpp gradient [55,56]. This expansion provides the necessary scaling. However, Zhu et al. challenged Pent’s role as the primary global expander due to its limited spatial range [57]. They instead proposed a pseudo source-sink model, demonstrating that scaling is driven primarily by the Dpp-mediated feedback down-regulation of its own receptors, rather than by a secreted expander. Another mechanism has been suggested by [58], who propose that Dpp gradient scaling is driven by a tunable ‘recycling gear,’ where the feedback regulator Pent modulates receptor binding kinetics to favor the re-exocytosis of internalized ligand over its lysosomal degradation. By progressively increasing the fraction of Dpp returned to the extracellular space, this mechanism actively extends the morphogen's effective range to match the growing tissue size.

### Drosophila pupal wing

Unlike Dpp signaling in the *Drosophila* embryonic and larval development, pupal wing Dpp signaling lacks a continuous 2D gradient and instead transitions between planar and localized 3D Dpp distributions [18]. Instead, it relies on localized ligand distribution through restricted extracellular diffusion from discrete, vein-associated sources, where the ligand is locally captured in crossvein (CV) territories through shuttling [8,19,59]. During this stage of development, Dpp signaling promotes the formation of the LVs and the CVs [7]. While LV precursor cells are first defined during larval development, the patterning of the CVs occurs during the pupal stages [7]. At this stage, Dpp expression expands into the LVs, where it acts locally to refine LV fate and at a long range to direct CV formation [7]. The formation of the posterior crossvein (PCV) is a result of Dpp expression in adjacent LVs, whereas the anterior crossvein (ACV) is formed when Dpp is expressed in a stripe intersecting that region [7].

Dpp signaling in the pupal wing regulates tissue proliferation following a transition from lateral diffusion within the LVs to interplanar diffusion between epithelial layers [7,19,20,60]. There are three main stages during pupal development (first apposition, inflation, and second apposition), which take place at 8, 10–20, and 20 h after pupal formation (APF), respectively. During the first apposition, Dpp is expressed in the LV primordial cells (LV2–5). Dpp maintains a tissue-wide gradient through lateral diffusion during inflation (∼18 h APF) and regulates tissue proliferation by inhibiting *brk* expression [20,60]. During the second apposition stage (∼24 h APF), Dpp signaling becomes localized to the LVs, diffuses vertically between the dorsal and ventral epithelia, and undergoes long-range diffusion from adjacent LVs into the PCV region to facilitate PCV formation and the formation of the 3D tissue architecture [7,20,60].

Paralleling the facilitated transport mechanisms described in the *Drosophila* embryo, extracellular regulators including Sog and Tld-related proteases, shape Dpp signaling in the pupal wing [7]. However, the Tolloid-related (Tlr) protease involved in PCV formation in the pupal wing, Tlr, exhibits slower Sog cleavage kinetics (five-fold) than its embryonic counterpart Tld and performs a non-redundant, tissue-specific function [32,61]. Transient Dpp expression is thought to be stabilized by Dpp signaling in the anterior lateral region of the pupal wing through the positive feedback factor, Crossveinless-2 (CV-2) [7,62]. CV-2 is suspected to promote local Dpp signaling by facilitating ligand retention and spatial redistribution [7].

While quantitative models describing the Dpp gradient have been developed for the *Drosophila* embryo and larval wing, no quantitative models currently exist that describe Dpp signaling in detail in the pupal wing, likely due to the discrete and transient nature of Dpp sources at this stage. Instead, Dpp signaling in the pupal wing has been primarily described through conceptual models involving restricted extracellular diffusion and shuttling ligand redistribution mediated by extracellular regulators such as Sog and Tld-family proteases. Additionally, it is suspected that there is a feedback mechanism between LV and PCV formation that facilitates the vectorial transport of Dpp from LVs to the PCV. This existence of feedback is supported by disrupted BMP signaling when LV Tkv receptors are constitutively active and increased LV Dpp motility and improper vein formation when Tkv receptors are disrupted [60].

### Drosophila germline stem cells

Dpp signaling is required for the self-renewal of GSCs and is down-regulated to allow GSC differentiation into cystoblasts (CBs) [21]. The GSCs are located at the apical tip of the germarium, within the GSC niche, which is composed of the terminal filament cells, cap cells (CpCs), and anterior escort cells (AECs). JAK/STAT signaling in the CpCs [63] and Wnt signaling in the AECs [64] and Hedgehog release from cap cells produce Dpp [65], such that the Dpp originating in the CpCs is the major source of the ligand in GSCs [12,66]. The CpCs being at the apical tip of the germarium ensures a higher level of Dpp is available to the GSCs than the CBs [12].

The distribution of Dpp within the GSC niche is shaped by components in the ECM, including heparan sulfate proteoglycans (HSPGs) and Type IV collagens, which ensure Dpp is contained in the niche and concentrated near the cap cells [67]. The HSPGs, for instance, the glypican encoded by Dally, are expressed in the cap cells and bind to Dpp to restrict its diffusion and stabilize it [68]. Dally-bound Dpp forms a reservoir of Dpp near the cap cells and is a major source of ligand to the GSCs, accessed through its actin- and microtubule-rich cytoneme projections [12]. Note that the microtubule-rich cytonemes up-regulated in response to BMP signaling also attenuate BMP signal transduction, enabling dynamic modulation of BMP signaling after GSC division [12]. Type IV collagens, including Viking (Vkg) and Dcg-1, also bind to Dpp and restrict it within the niche, such that the germaria in *vkg* mutant flies have an increased number of GSCs [69]. In addition, Tkv functions in the ECs, independently of BMP signaling in the GSCs, as a receptor sink to limit Dpp to the niche.

Further, a number of signaling pathways, including the Wnt, epidermal growth factor receptor (EGFR), and mitogen-activated protein kinase (MAPK), work in conjunction to restrict Dpp to the GSC niche. The Wnt signaling pathway, specifically Wnt4, which is expressed in a graded fashion: higher in the TFs and CCs and lower in the posterior escort cells, is activated downstream of BMP signaling in posterior escort cells [71,72]. The disruption of Wnt4 and other components of the Wnt pathway results in an increased number of GSC-like cells, preCBs, and CBs [73]. Factors such as aging also affect the Wnt activation, such that as the flies age, Wnt activation in the cap cells increases and in escort cells decreases, whereas *dpp* expression exhibits an opposite pattern: lower in the cap cells and higher in escort cells [73]. The GSCs and ECs engage in cross-talk such that GSCs promote EGFR–MAPK pathway activation in the ECs to repress *dally* expression, thus restricting Dpp to the GSC niche [68].

In addition, several intracellular feedback mechanisms, including the positive feedback mediated through fused (Fu) [74–76] and the negative feedback mediated through daughters against Dpp (Dad) [76–78], ensure Dpp signaling is up-regulated in GSCs and down-regulated in the CBs that are located only one cell diameter away to precisely control stem cell differentiation on a two-cell diameter length scale.

### Zebrafish

During zebrafish embryogenesis, secreted BMP2 and BMP7 homo- and heterodimers are secreted by a minority of cells [79]. The extracellular regulatory network sharpens the spatial distribution and effective activity of the dimeric BMP ligands to establish a gradient across the zebrafish embryo, characterized by high ligand concentration on the ventral side and progressively lower levels toward the dorsal side. These ligands interact with and recruit cell membrane receptors into signaling complexes that transduce and spatially pattern Smad activation within cells. A diverse set of extracellular regulators plays a crucial role in regulating this gradient, including antagonists Chordin (homolog of *Drosophila* Sog), Noggin, and Tsg, which bind BMP ligands and limit their diffusion [80–83]. Other modulators such as Tld, CV-2, and sizzled influence ligand stability, degradation, and shuttling [80–83]. These regulators contribute to counter-gradients, extracellular shuttling, and transcriptional mechanisms to enhance the overall robustness of the gradient formation (Figure 3D,F). More recent characterization of this gradient through quantification through the development of a computational model has indicated that the source-sink mechanism (Figure 3E) is the most likely explanation for gradient formation, with the ventral BMP ‘source’ and dorsally enriched Chordin as the ‘sink’ that binds and redistributes BMP ligands [79,84]. A critical aspect of this patterning is scale invariance: the ability to maintain proportional patterning when the size of the embryo changes. In zebrafish, the BMP signaling gradient maintains scaling despite experimental reductions in embryo size of up to 30%, a robustness achieved by active feedback regulating the stability of the Chordin sink [35]. Li et al. have since generated a model that utilizes complex three-dimensional virtual embryos and simulated an advection–diffusion–reaction using partial differential equations (PDEs) to more closely mimic the geometry of the system, which further supported the source-sink mechanism [85]. However, limitations of this model derive from lower accuracy in experimentally quantified gene expression domains due to their significant impact on model outputs.

### Human stem cells

*In vitro* models such as micropatterned hESC/hiPSC colonies mimic peri-gastrulation-like patterning using BMP4 stimulation. When colonies are stimulated with uniform BMP4, they self-organize into a radial gradient of SMAD1/5/8 activity [86]. Inhibitory signals restrict BMP4 activities to the colony periphery and generate an activin/nodal gradient toward the interior that patterns mesendodermal fate. Interestingly, BMP-SMAD signaling exhibits an asymmetric gradient in the post-implantation amniotic sac embryoid, which is suspected to play a role in tissue patterning. During pre-gastrulation, there is a prominent population of nuclear pSMAD1/5 at amniotic poles, and the nuclear pSMAD1/5 is present in the tissues before the transcription factor, CDX2, involved in differentiation and cell proliferation [87].

Quantitative computational models, including reaction–diffusion frameworks [88], agent-based models focused on gene regulatory networks (GRN), cell–cell interactions [89], and cross-signaling models of the BMP-Wnt-Nodal cascade [90,91], have shown how colony geometry, inhibitor feedback, and signaling dynamics collectively shape the BMP-SMAD gradient. These modeling studies demonstrate that the patterning in hiPSCs, along with other developmental systems, arises from the pattern developed from a self-organized BMP/Smad signaling gradient, and its formation can be accurately captured and predicted through computational approaches. Furthermore, these modeling studies can be used to improve differentiation efficiency toward mesodermal and endothelial cells [92,93].

Many differentiation protocols aiming to recapitulate the natural development of specific cell types utilize concentration gradients as a screening tool to identify a singular optimal concentration, revealing the critical thresholds required for germ layer and subsequently lineage specification. For instance, the use of orthogonal gradients of BMP2 and adhesion peptides allows for identification of specific coordinates that determine the most efficient concentration combinations for differentiation, as seen in osteoblast fate specification [94]. This quantitative sensitivity is also a defining feature of mesodermal branching. With low levels of BMP4 in combination with Activin A, optimize the induction of cardiogenic mesoderm, and higher BMP4 concentrations pivot the population toward a hemogenic fate [95]. While these studies focus on global optimization, labs utilizing micropatterned environments demonstrate that these same thresholds drive spatial ordering. In Yang et al., the geometric confinement established by the micropatterned surface influenced cell growth in colonies and established a radial BMP4 gradient. The high concentrations at the colony edges influence a non-neural ectoderm fate, while the low BMP environment of the center permits neural ectoderm differentiation [96]. This confirms that the spatial organization of the embryo is a dynamic response to the local signaling dose, independent of the initial physical constraints of the tissue.

## Intracellular Smad dynamics and signal processing

On the cellular level, BMP signaling is interpreted through the dynamic behavior of intracellular Smads, which acts as the core signal-processing module of the pathway. The Type II BMP receptors are constitutively active, and the GS-transmembrane domain on the Type I receptor is activated by the formation of the ligand–receptor complex. Following ligand-induced Type I receptor activation, Smads (Mad in *Drosophila*; Smad1/5/(8/9) in vertebrates) are phosphorylated at the two C-terminus serine residues on the R-Smads, which then form homo/hetero-dimeric and trimeric complexes with the common Smad (Medea/Smad4) to enable nuclear translocation and transcriptional regulation [97]. Importantly, Smad signaling is inherently dynamic: Smad complexes continuously shuttle between the cytoplasm and nucleus and are subject to multiple layers of regulation at the molecular level, including inhibitory Smads, phosphatases, and ubiquitin-mediated degradation.

The eight vertebrate Smads can be classified as TGFβ R-Smads (Smad2 and Smad3), BMP R-Smads (Smad1, Smad5, and Smad8/9), I-Smads (Smad6 and Smad7), and a Co-Smad (Smad4). The R-Smads and Smad4 share two highly conserved protein domains, including the N-terminal Mad homology (MH)-1 and C-terminal MH2 domain, which are separated by a less conserved serine and proline-rich linker region [98]. The MH1 domain allows for DNA binding (except to Smad2) and nuclear import, and the MH2 domain regulates Smad interactions with other Smads, transcription factors, co-activators, and co-repressors. Post-translational modifications, including sumoylation, ubiquitination, acetylation, phosphorylation, and PARylation occur at the two MH domains or the linker to regulate Smad activity or its interaction with other signaling pathways through MAPKs and CDKs [99].

Inhibitory Smad activity (e.g., Dad in *Drosophila* [12,78] and Smad6/7 in vertebrates [88–91]) is directly induced by BMP signaling and suppresses further R-Smad phosphorylation through inhibition of the Type I receptor. This forms a negative feedback loop that prevents cells from prolonged or excessive signaling. Alternatively, phosphatases, including Dullard/CTDNEP1, regulate this pathway by dephosphorylating the phospho-Mad complexes or the BMP receptors (in *Xenopus*) [26,104] to synergistically promote signal termination in conjunction with the I-Smads [27]. Ubiquitin-mediated degradation provides an additional layer of control over BMP-SMAD signaling by limiting signal duration and amplitude. For instance, E3 ubiquitin ligases (SMURF1/2) target activated receptors and phosphorylated Smads for proteasomal degradation [105]. These processes shape the amplitude, duration, and timing of nuclear pSmad signals, allowing cells to integrate BMP inputs over time and preventing misregulated pathway activation.

Across all the model organisms described, phosphorylated Smad1/5/(8/9) (pSmad) in vertebrates and phosphorylated Mad (pMad) in *Drosophila* serve as the primary quantitative readouts of the BMP pathway [106–108]. Their nuclear localization provides a robust and experimentally accessible proxy for ligand–receptor engagement, signal transduction, and transcriptional output. Because secreted Dpp/BMP ligands are difficult to directly visualize due to their complex extracellular regulatory interactions, intracellular Mad/Smad protein phosphorylation (pMad/pSmad) is used as a functional reporter of pathway activation. Immunofluorescent staining followed by the confocal microscopy enables quantification of nuclear pMad/pSmad intensity, providing a readout of spatial distribution of BMP signaling patterns across tissue without requiring absolute molecular calibration [35,109].

In zebrafish and *Drosophila*, pSmad/pMad immunostaining with phospho-specific antibodies is done at various stages of developmental systems. In zebrafish embryos, analysis relies almost exclusively on fixed immunostaining, due to the rapid gradient formation (3–9 h post-fertilization) that governs the dorsoventral patterning. Within this limited time frame, the embryo develops from hundreds to thousands of cells, which makes live tracking and imaging of BMP signaling technically challenging. Although live imaging for other transcription factors has been targeted, pSmad immunostaining remains the most robust and widely favored BMP-quantification method in zebrafish embryos [35,73,110,111]. In *Drosophila*, pMad immunostaining is broadly applied across germline stem cells (GSC-preCB), ovaries, third instar larvae, the pupal wing, and wing discs to approximate BMP signal transduction in both wild-type and mutant contexts [27,75,112]. The pMad immunostaining in the ovaries dissected from 3–5-day-old adult flies has facilitated the quantification of pMad asymmetry during the G1/S phase of GSC division [27]. Likewise, in the embryos, nuclear pMad immunostaining has found that the pMad domain typically spans 5–6 cell diameters and that in *sog* mutants, uniform distribution of Dpp in the dorsal region resulted in a broadened pMad pattern spanning 10–13 cell diameters [43,44,46]. More recently, live imaging approaches have begun to complement fixed pMad measurements. In *Drosophila* embryos, nuclear accumulation of Smad4/Medea has been quantified *in vivo* and reveals a strong correlation between pMad levels and nuclear Med-GFP intensity [16]. These findings suggest that nuclear Medea can be used to serve as a live proxy for BMP activity [16,17]. Since pSmad/pMad patterns encode both spatial and temporal features of BMP signaling, they serve not only as a measurement tool but also as the central benchmark for linking and validating experimental datasets with diverse mechanistic and data-driven models that aim to explain gradient formation, signal interpretation, and downstream gene regulation across these systems.

## Interpretation of BMP signals via transcriptional regulation

Despite differences in ligand–receptor composition and extracellular modulators, the intracellular Smad-mediated signaling logic downstream of BMP remains remarkably conserved. Understanding how this conserved signaling machinery translates intercellular BMP gradients into precise target gene expression patterns provides a critical link between molecular signaling mechanisms and systems-level developmental outcomes. Across these systems, the BMP activity is interpreted through a tiered transcriptional response of downstream target genes. This network includes both direct activators and negative regulators, which collectively shape the signaling outcome by implementing feedback mechanisms that modulate pathway activity and influence systems-level behavior.

### Drosophila embryo development

BMP signaling in *Drosophila* embryos directs downstream target genes that have been classified into three types—Type I, II, and III—based on their threshold sensitivities to BMP signal strength and their spatial expression patterning [113]. Several studies have illustrated these distinctions by examining how spatial domain of gene expression shifts in wild-type and mutant embryos [7,23,47,114].

Type I genes are induced only under high BMP signaling at the dorsal midline. Type II genes require moderate BMP input and are expressed in an intermediate band. Type III genes are the most sensitive and respond to low levels of BMP signaling. These genes are activated early and often maintain broad expression after the BMP signal refines to a narrow dorsal stripe of 3–4 cells, suggesting a memory mechanism may be at play [16]. Consistent with this, Brantley et al. observed that the *zen* protein that has a gradual, spatially broad accumulation before gastrulation formed a broad distribution exceeding the width of the Med-GFP gradient across the dorsal surface [17]. Zen may act as an early ‘sensitizer’ that helps cells respond to low BMP, broadening or accelerating the transcriptional response [17]. These findings illustrate the spatiotemporal control of BMP signaling required for precise patterning in early *Drosophila* embryos.

Another study revealed that BMP signaling modulates the frequency of transcriptional bursts—periods when a gene is actively transcribed—rather than the speed of transcription once active by measuring *ush* (II) and *hnt* (I) in the embryo. In lower BMP regions, both genes showed reduced promoter occupancy and burst frequency due to a lower rate of promoter activation, while the transcriptional loading rate and burst duration remained unchanged [115].

### Drosophila larval wing disc

In the larval wing disc, the pMad-Medea complex drives activation of target genes such as *optomotor-blind (omb), dad*, and *spalt major (sal)* and represses *brinker (brk)*. *Omb* and *sal* are transcription factors that promote the transcription of genes involved in wing formation and patterning, whose expression scales with BMP signal strength, while Dad directly inhibits BMP signaling through a negative feedback loop and Brk suppresses BMP target gene expression. *Brk* acts as a transcriptional repressor whose expression is inversely proportional to Dpp signaling and is regulated by pMad-mediated transcriptional repression [116]. The transcription of these target genes is both dependent on *brk* expression (Brk-mediated repression) and pMad/Medea complex levels (pMad/Medea-mediated repression and activation). Brk-mediated repression blocks promoters or inhibits activators to repress target gene expression. This repression can occur through recruitment of the co-repressors, such as Gro and CtBP, or can act independently of co-repressors, as it does when modulating *omb* and *sal* expression [48,117]. The pMad-mediated transcriptional repression and activation occur when the pMad/Medea complex recruits distinct co-factors to bind to silencing and activating elements, respectively [118]. The complex recruits the co-repressor Schnurri (Shn), which suppresses *brk* transcription by binding to a *brk* silencing element and silencing its locus, thereby relieving Brk-mediated repression of target genes (derepression) [48].

Expression of *dad* is primarily controlled by direct activation by the pMad/Medea complex, whereas *omb* can be activated through relief of Brk-mediated repression and does not strictly require direct pMad/Medea input, while *sal* requires both [119]. While *omb* does not require pMad/Medea activity, input from pMad/Medea can enhance its expression [119]. Similarly, relief of Brk-mediated repression can enhance *dad* expression, although it is not required for its activation [119]. High and intermediate levels of Dpp promote *dad* expression and allow for pMad/Medea-mediated transcription suppression of *brk*, which prevents Brk-mediated repression. This facilitates the activation of *sal* and *omb*, thereby increasing BMP transcriptional output, which is modulated by Dad-mediated negative feedback. At low Dpp levels, limited pMad/Medea complex activity limits *dad* expression and allows for Brk-mediated repression of *sal* and *omb* expression to occur. The repression of *sal* and *omb* reduces BMP transcription output [48,116]. This Dpp-dependent transcription guides proper adult wing shape and vein formation.

Several models have been developed to explain how transcriptional regulators, including negative regulators like Brk and Dad and positive regulators such as Vestigial (Vg), are integrated at the tissue level to stabilize the Dpp gradient and coordinate robust growth through feedback mechanisms. Two main frameworks, growth equalization and Vg feed-forward models, have been proposed to explain how these feedback mechanisms balance proliferation across the wing disc [48]. In the growth equalization model, Brk acts as a growth suppressor that is inversely regulated by Dpp activity [48,120]. This model predicts that Dpp activity is highest in medial cells and declines laterally [48,120]. The growth equalization model also predicts that if Dpp signaling were artificially uniform across the tissue, lateral proliferation would increase due to reduced Brk expression [48,120]. However, this prediction contradicts *in vivo* findings that demonstrate Brk levels are normally high in lateral regions, where they suppress Dpp/BMP pathway activity and reduce proliferation [48]. The Vg feed-forward model postulates that coordinated input from Vg and the morphogens Dpp and Wg creates a feed-forward loop that recruits adjacent non-wing cells to become proliferating wing cells [121]. In this model, Dpp and Wg induce Vg expression; Vg then cooperates with Yorkie to reinforce Vg expression in adjacent cells, thereby expanding wing cell development through a positive feedback loop [48,121]. Importantly, these models highlight that tissue growth is tightly regulated by Dpp signaling and its transcriptional outputs, which coordinate proliferation and cell recruitment across the wing disc [122]. While these models primarily describe how Dpp signaling regulates tissue growth, additional mechanisms, such as the expansion-repression model, describe how tissue growth impacts gradient formation through Dpp scaling (in the ‘Mechanisms of extracellular BMP distribution and receptor-level regulation’ section) [55,120].

### Drosophila pupal wing

The core mechanisms of the BMP signaling pathway described in the *Drosophila* larval wing disc are conserved during the pupal stage of development; however, the downstream transcriptional targets differ. As a direct output of the pMad-Medea complex, Dad is the only conserved downstream transcriptional target between the larval and pupal stages [5,6]. The other direct downstream target of the BMP pathway during pupal development is the BMP-responsive extracellular regulator, CV-2 [7,8]. CV-2 is an extracellular regulator of the BMP pathway that is suspected to act in a biphasic manner where it promotes BMP signaling at low Dpp levels and inhibits BMP activity at high Dpp levels [59]. The biphasic manner of CV-2 is supported by findings where ectopic BMP signaling resulted in high levels of CV-2, and mutations in CV-2 prevented local BMP signaling in the PCV during pupal development [59].

Other downstream targets of the BMP pathway during pupal development occur indirectly through cross-talk with the EGFR/MAPK and Notch pathways. These indirect downstream targets of the BMP pathway facilitate vein differentiation through the expression of *Rhomboid (rho), Star (s)*, and *argos (aos)* and the repression of *blistered/Srf (bs)* [9–11] in the EGFR/MAPK pathway. EGFR signaling is amplified by *Rho* and *Star* expression, which are integral in vein formation, since they are highly expressed in L3 and L4 proteins, where Dpp levels are amplified in the pupal wing [9]. *Aos* is an inhibitor that interacts with the EGFR pathway through a negative feedback loop [9]. During pupal development, the EGFR pathway represses *bs* expression, which is lost in the proveins and retained in the interveins [9,11]. Inactivation of the EGFR pathway disrupts vein formation in the pupal wing; in particular, loss of *rho* and *star* blocks distal vein formation but has little effect on the LVs and CVs [9]. The Knirps/Knirps-like transcription factors (Kni/Knrl) induce EGFR activation in the L2 pro-vein through *rho* transcription by transducing BMP pathway positional information. Development of the CVs in the pupal wing is first initiated by BMP signaling but is maintained until adulthood through the EGFR pathway upon *rho* and *star* transcription, likely caused by BMP pathway cues. Ectopic vein formation is a result of pathway cross-talk between the EGFR, BMP, and Notch pathways, where hyperactivity of *rho* and *star* interacts with Dpp to activate BMP signaling, which then activates the Notch pathway through its ligands, *Delta* and *Serrate* [9]. Loss of one of these pathways reduces activity of the other pathways, demonstrating their interaction and functional positive feedback between the two [9]. Yan et al. propose a positive feedback model that requires the co-activation of the EGFR and Dpp pathways for pupal vein differentiation [123]. Co-activation of these pathways promotes ligand production from each pathway, which reinforces signaling through a positive feedback loop [123]. This system demonstrates bistability, where co-activation in the pro-veins produces a stable ‘on’ state, and down-regulation of either pathway results in switching to an ‘off’ state, characterized by reduced pMad and dpERK levels [123].

### Drosophila germline stem cells

In the *Drosophila* ovary, BMP signaling in the germline stem cell niche maintains GSC identity through differential gene expression. This is achieved through repression of *bags of marbles* (*bam*), a driver of germline differentiation. Asymmetric division moves the differentiated daughter CB away from the niche, reducing BMP exposure and hence pMad levels; with the loss of BMP-mediated repression, *bam* is unrepressed in the CB, initiating differentiation. Genetic perturbations that maintain high pMad in daughter cells prevent *bam* expression and block differentiation, underscoring the necessity of down-modulating BMP after niche exit [124,125]. The sharp pMad-*bam* axis is maintained by intermediaries including a serine/threonine kinase Fu, along with E3 ligase Smurf, which targets BMP receptor Thickveins for ubiquitination in the CBs [74].

Further, Wilcockson and Ashe performed RNA-Seq on genetically expanded GSC populations—flies carrying constitutively active *tkv* together with *bam* knockdown—allowing comparison of ‘high Dpp’ and ‘low Dpp’ GS [12]. In the high Dpp condition, pMad^+^ GSC-like cells with single and round spectrosomes were observed throughout the germarium, reflecting global activation of the self-renewal program. In the low Dpp condition, *bam* knockdown limited differentiation, which again led to an abnormal increase in pMad-GSC-like cells with single and round spectrosomes across the germarium. This work identified ∼300 Dpp-responsive genes, including *dad, Rfx*, and *futsch*, thereby broadening the catalog of BMP targets in germline stem cell regulation.

Intracellular regulation, mediated by phosphatases and feedback loops, sharpens this transition to establish pMad asymmetry. The GSC division cycle has a duration of 12–15 h, during which the GSC and preCB have a shared cytoplasm [126]. The distribution of pMad in the G1/S phase of cell division is asymmetric, such that pMad expression in the preCB is less than the pMad concentration in the GSC. Mad phosphatase, Dullard, dephosphorylates Mad at the nuclear pores, which, in conjunction with higher BMP activation in the GSCs than CBs, establishes pMad asymmetry [27]. Further, *Dad* up-regulated BMP signaling within the GSC limits, receptor-mediated phosphorylation of R-Smads, and targets Type I receptor Tkv for ubiquitination [12,78]. This negative feedback likely enforces the steep signaling boundary between GSCs and CBs, minimizing ‘leakage’ of stemness signals into differentiating daughter cells. This network ensures that only cells in proximity to the niche retain stem cell identity, while even modest decreases in BMP signaling in daughters reliably trigger *bam* expression and differentiation. This spatially gated, robust switch safeguards the balance between self-renewal and differentiation.

Several modeling works have focused on simulating the bistable nature of the GSC/CB differentiation. Pargett et al. developed a model of BMP signaling in the germarium to identify the role of Brat as a differentiation factor [127]. Their simulations suggested that Pum-Nos-mediated degradation of Brat in GSCs preserves self-renewal, while in CBs, the absence of this repression allows Brat to antagonize BMP signaling and promote differentiation. Xia et al. modeled the experimentally derived reciprocal antagonism as a positive feedback loop between Fu and the BMP pathway, showing that Fu repression within GSCs sustains BMP signaling to maintain stemness, whereas Fu accumulation in CBs facilitates pathway down-regulation and differentiation [75]. More recently, Shaikh and Reeves developed a biologically informed mathematical model of multi-compartment GSC division to investigate the dynamic roles Dad and Fused play in determining cell fate [128]. Their analysis revealed that Dad optimally tunes BMP signal transduction to maintain GSC homeostasis, and in *Dad* knockout mutants, GSCs were more likely to divide symmetrically. Their work identifies the synergistic role of Dad and Fused, rendering robustness to stem cell division.

### Zebrafish

In the developing zebrafish embryo, cells have been shown to interpret the BMP gradient through a concentration threshold [13]. Distinct levels of phosphorylated Smad5 (pSmad5) activate specific sets of target genes, creating sharply defined expression domains across the embryo. Using transcriptional profiling combined with single-cell pSmad5 measurements, Greenfeld et al. identified over fifty primary BMP target genes and demonstrated that cells decode BMP signaling based on absolute pSmad5 concentration cutoffs rather than relative position or temporal history of exposure [13].

Similar to the *Drosophila* embryo target genes, these BMP target genes can be grouped into three categories according to their activation thresholds and spatial expression profiles along the dorsoventral (DV) axis. High-threshold genes, such as *sizzled* (*szl*) and *tp63*, are expressed exclusively in the most ventral regions where BMP signaling is strongest. Intermediate-threshold genes, including *foxi1* and *gata2a*, are activated in the ventral-animal domains at moderate pSmad5 levels. Finally, low-threshold genes such as *bambia* (or *bambi*) respond to weaker pSmad5 activity closer to the dorsal side. Together, these categories represent discrete transcriptional responses corresponding to specific concentration steps within the BMP gradient, suggesting that pSmad5 functions as a digital readout gated by defined concentration thresholds [13,129].

Several of these BMP target genes also participate in feedback mechanisms that fine-tune the pathway. *Bambia* (low-threshold) encodes a transmembrane pseudoreceptor that attenuates BMP signaling by acting as a negative regulator of the pathway in the already low-activity area [130]. Similarly, *szl* (high-threshold) encodes a secreted inhibitor that prevents Chordin (Chd) cleavage by Tld, thereby maintaining the BMP gradient; *szl* also possesses both BMP and Wnt inhibitory activity [81,131]. At the same time, high BMP activity represses *chd* transcription, confining its expression to the dorsal regions. Since these feedback loops become active after gastrulation onset, they serve primarily to stabilize and regulate the established BMP signaling domains rather than to initiate them. A computational model of this system from Tuazon et al. in 2020 is one of the few that consider transcription, which makes the network considerably more complex [132]. Their model strongly supported the source-sink mechanism under comparison with the wild-type and a variety of mutant lines [132].

Beyond this threshold mechanism, BMP target gene expression is refined through combinatorial and temporal inputs from other signaling pathways. Cross-talk with FGF and Nodal signaling helps sharpen domain boundaries; inhibition of either pathway collapses the diversity of BMP target expression into uniform ventralized patterns [84,132,133]. In parallel, extracellular modulators such as Chd, Bmp1a, and Tld sculpt the BMP gradient by restricting Chordin diffusion and establishing localized ligand sinks. Together, these interactions ensure the precision and robustness of BMP-mediated dorsoventral patterning in the zebrafish embryo.

### Human pluripotent stem cells

In human pluripotent stem cells, including hESCs and hiPSCs, some of the most immediate and conserved BMP targets are the inhibitor of differentiation (ID) genes ID1–ID3 [15,134,135]. ID proteins interfere with factors that promote neural identity, preventing premature activation of the neural program and helping cells remain undifferentiated [135–139]. In both mouse and human pluripotent systems, ID proteins serve as key mediators translating SMAD signaling into transcriptional and epigenetic control of pluripotency and lineage bias [140]. ETV2 and ETS2 are important transcription factors that work with the BMP pathway to regulate hiPSCs differentiation into mesodermal and endothelial cells [141,142].

BMP signaling also activates a multilayered negative-feedback network that tightly regulates the duration and intensity of the pathway at both intracellular and extracellular levels. The inhibitory SMADs, SMAD6 and SMAD7 are transcriptionally induced by BMP-SMAD complexes and subsequently inhibit receptor activation by competing with R-Smads or by recruiting E3 ubiquitin ligases (SMURF1/2) to promote receptor and R-SMAD degradation [100–103].

Similar to its role in zebrafish, BAMBI (BMP and activin membrane-bound inhibitor), a kinase-deficient pseudoreceptor, is up-regulated by BMP signaling and inhibits further receptor complex formation, adding another layer of intracellular negative feedback [143–145]. At the extracellular level, BMP signaling also induces several secreted antagonists, including NOGGIN, CHORDIN, and follistatin [146–148]. These factors sequester BMP ligands and restrict their diffusion by competitively binding them, thereby establishing a complementary feedback circuit that modulates BMP availability at the ligand–receptor interface [149,150].

## Computational modeling approaches that drive mechanistic and cross-species insights

**Figure 4:**
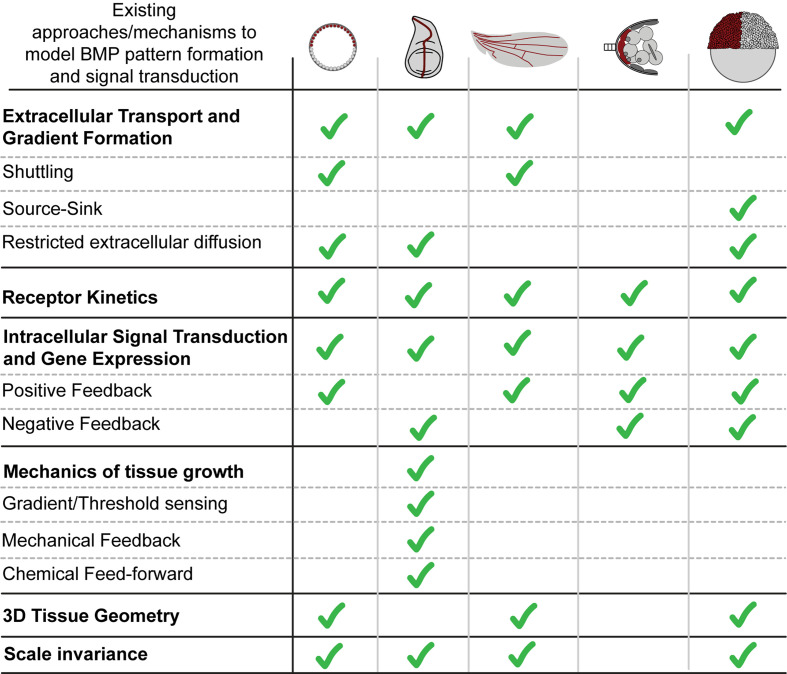
Overview of mechanistic features represented in existing mathematical models of BMP gradient formation, signal transduction and gene expression across the reviewed model systems Dashed lines indicate that all the mathematical models listed for a given model system incorporate a common mechanistic component. Checkmarks indicate that at least one published computational or mathematical model for that system explicitly includes the corresponding mechanism. The absence of a checkmark does not imply that the mechanism is biologically absent, but rather that it has not been explicitly incorporated into the models summarized in Table 2. This demonstrates that, despite spanning diverse systems, all of the models have shared mechanistic foundations. Table 2 provides a detailed listing of the specific models associated with each system.

Building on the system-specific models reviewed above, including pattern-formation models, bistability and GRN models, feedback-control frameworks, and source-sink transport models, the computational approach can be summarized into several major categories: Intracellular ODE models capture receptor activation, Smad phosphorylation, nucleocytoplasmic shuttling, and feedback loops and help explain gene-regulatory mechanisms (e.g., bistability in stem cell systems, signal amplification, and buffering) observed across species [27,75,76,112,127]. PDE models based on reaction–diffusion equations extend these insights to the tissue scale, showing how extracellular transport, inhibitor gradients, ligand shuttling, and transcriptionally induced modulators shape the pSmad/pMad patterns underlying dorsoventral patterning, germline maintenance, wing growth control, and self-organization in human pluripotent stem cells [40,46,47,79,84,85,88]. Complementing these frameworks, agent-based and GRN models integrate cell–cell interactions, spatial neighborhood effects, and transcriptional networks to explain how local signaling generates colony-scale or tissue-scale pSmad/pMad heterogeneity [89]. Stochastic models highlight how intrinsic noise in ligand–receptor binding or transcription contributes to variability in pSmad/pMad readouts [127,151]. Multi-scale and mechanochemical models combine intracellular biochemistry with tissue geometry, cell flow, or morphogenesis, reflecting the complex environments in which BMP gradients are interpreted [47,85,91]. To place these system-specific models in a unifying framework, Figure 4 summarizes the dominant mechanisms of BMP gradient formation, SMAD signal transduction, and gene regulation across all reviewed systems, and Table 2 catalogs the corresponding computational models.

**Table 2:** Computational Models describing the behavior of the six systems

System	Modeling approach/type	Key finding or purpose
*Drosophila* embryo	**Shuttling and receptor-mediated degradation** [24]	Showed that the sharp pMad peak requires the Sog/Tsg shuttling mechanism and Tolloid-mediated cleavage.
	**Extracellular shuttling** [45]	Established the first computational framework for dorsal-ventral patterning to demonstrate the plausibility of the Sog-mediated shuttling mechanism.
	**Positive feedback** [42]	Proposed that positive feedback and bistable dynamics reinforce and maintain sharp signaling boundaries.
	**Receptor kinetics and feedback** [41]	Showed that slow, reversible receptor–ligand binding kinetics provide robustness against changes in receptor levels.
	**3D geometry** [43]	Argued that robustness must be evaluated by its effect on threshold and downstream gene expression.
*Drosophila* wing disc	**Restricted diffusion** [45]	Investigated competing hypotheses to show that Dpp transport occurs via restricted extracellular diffusion.
	**Gradient** [128]	Proposes that the slope of the Dpp gradient drives cell proliferation if the slope is steeper than a certain threshold.
	**Threshold (gene expression)** [48]	Proposes that cells proliferate if Dpp signaling is above a certain threshold level.
	**Temporal** [54]	Proposed that cells respond to a relative increase (not absolute level) in Dpp to trigger uniform proliferation.
	**Growth equalization model** [45,48]	Modeled how the target gene *brk* acts as a growth suppressor to balance proliferation across the disc.
	**Mechanical model** [129,130]	Modeled how physical tissue forces (compression/stretching) provide feedback to ensure uniform proliferation.
	**Vg feed-forward model** [131]	Modeled how Dpp, Wg, and Vg interact in a feed-forward loop to coordinate and expand the wing tissue.
	**Expansion-repression model** [50]	Modeled how Pent acts as a rapidly diffusing ‘expander’ molecule of the Dpp gradient.
	**Pseudo source sink** [52]	Modeled how Dpp-mediated feedback down-regulation of its own receptors drives scaling.
	**Recycling** [53]	Modeled how Dpp gradient scaling is driven by a tunable ‘recycling gear’ mechanism where the feedback regulator Pentagone modulates receptor binding to favor ligand re-exocytosis.
*Drosophila* pupal wing	**Gradient formation** [21]	Describes Dpp gradient formation mechanism during first apposition, inflation, and second apposition of pupal wing development to create the 3D architecture of the adult wing.
	**Receptor-mediated degradation and shuttling** [8]	Discusses long-range Dpp transport through Sog/Ts2 shuttling mechanism and Tolkin protein-mediated cleavage. Proposed PCV formation is driven by Dpp/Gbb heterodimers.
	**Positive feedback loop** [8,55]	Discusses positive-feedback loop of the Sog/CV-2 mediated transport and its proposed role in releasing Dpp:Gbb heterodimers in LVs to facilitate PCV formation.
	**Bistability/positive feedback** [8,117]	Discusses the ability of this feedback loop to create sharp step-gradients for spatial bistability and how co-activation of the EGFR and Dpp signaling pathways are required for vein differentiation.
*Drosophila* GSC niche	**Bistability/differentiation** [98]	Modeled the GSC/CB switch to identify *Brat* as a key differentiation factor that antagonizes BMP.
	**Positive feedback loop** [61]	Modeled how *Fu* dynamics create a positive feedback loop to maintain stemness (GSC) or allow differentiation (CB).
	**Multi-compartment GSC division** [99]	Modeled how *Dad* and *Fu* work *synergistically* to ensure robust GSC division and homeostasis.
Zebrafish embryo	**Mechanistic/gradient formation** [65,70]	Tested theorized gradient models against mutant data, providing strong support for the source-sink mechanism.
	**3D simulation (advection diffusion reaction)** [132]	Developed a more geometrically accurate 3D model that also supported the source-sink mechanism.
	**Stochastic receptor model** [126]	Modeled receptor combinations for signal transduction, showing emergent low-pass filter mechanism arising from heterodimer–heterotetramer requirement.

These modeling strategies not only uncover the mechanistic principles driving BMP-SMAD patterning, which arises from the interplay of feedback regulation, extracellular transport, tissue geometry, and noise, producing robust and self-organized signaling landscapes across diverse biological systems, but also provide a common analytical language for comparing signaling behavior across the six systems in the present review. By enabling parameter inference, model transfer, and cross-species prediction, computational approaches facilitate a translational perspective in which conserved pathway logic can be distinguished from system-specific regulatory features. This perspective is exemplified by the ‘Digital Cousins’ framework, which demonstrates how a single mechanistic model, appropriately parameterized, can capture BMP signaling behavior across biologically distinct systems and reveal shared design constraints despite anatomical and molecular differences [152]. These integration approaches reveal how a conserved BMP-SMAD signaling module gives rise to diverse developmental outcomes across organisms, tissues, and scales.

## Pathway level performance objectives in system-specific contexts

**Figure 5:**
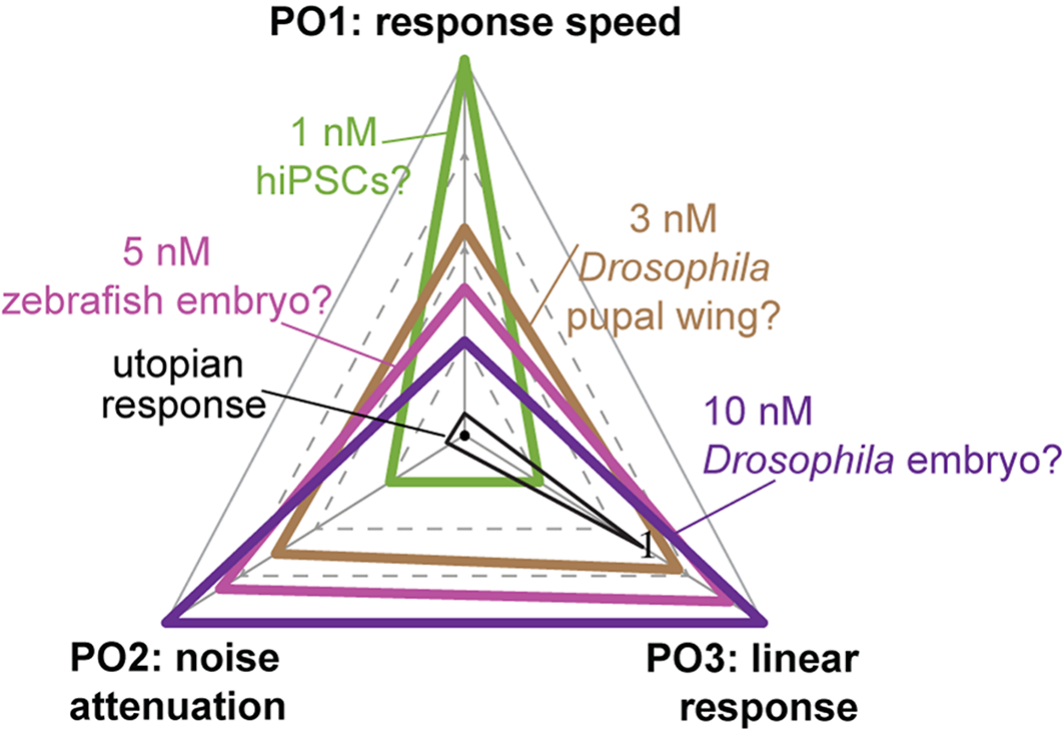
Performance trade-offs across model systems Simulation results from Shaikh et al. (2024) demonstrate the effect of varying phosphatase concentration (1 to 10 nM) on the balance of the POs, thus potentially allowing the highly conserved BMP pathway to exhibit diverse performance across model systems. In general, increasing phosphatase concentration decreases response speed and increases linear response while sacrificing noise attenuation. Embryo systems potentially favor response speed and linearity, while differentiating organs and stem cells favor noise reduction. The ‘utopian ideal,’ in which all three POs are optimized, such that POs one and two: response speed and noise attenuation, are minimum whereas PO3 is unity, is shown in black. The coordinates of the black marker are (0, 0, 0).

Despite the highly conserved nature of the BMP/Smad pathway across various organisms, its functional behavior varies strikingly across developmental and cellular contexts. Importantly, the diversity in pathway behavior does not arise from rewiring the core signaling network or altering the biochemical functions of its components, which are highly conserved. Instead, the diversity has been observed in the response speed, noise filtering capacity, and output sensitivity to pathway inputs, which represent competing POs [76]. These system-level behaviors can be quantitatively characterized by three minimal POs: (1) Response speed, indicated by the rise time (*t*_rise_), defined as the duration taken by the Smad signaling complex to reach 95% of its final steady state; (2) Noise filtering, indicated by the noise attenuation ratio, defined as the ratio of the coefficient of variation of the response signal to that of the input signal; and (3) Linear sensitivity with respect to varying extracellular inputs, indicated by the steady-state sensitivity coefficient, defined as the ratio of the fractional change in steady-state output to that of the input. For communication systems, an ideal or ‘utopian’ response would be characterized by fast response, high noise filtering capacity, and linear sensitivity to extracellular inputs. However, these POs for the BMP pathway are optimized to tune system-level behaviors that emerge from varying in non-conserved parameters (NCPs), such as the nuclear import rate of the Smad complex and the intracellular concentrations of Smad proteins and phosphatases, with cellular phosphatase levels exhibiting a strong influence. This tunability of the BMP pathway performance by varying NCP concentration, including phosphatase (Figure 5), was shown through simulations performed using a predictive BMP pathway model whose kinetic parameters were estimated by fitting to experimental data [37].

The balance among these performance objectives is system-specific, with each developmental or cellular context emphasizing different trade-offs (Figure 5). For example, in *Drosophila* germline stem cells, using the GSC cell-division model, analysis of the rise time in both wild-type and *dad* knockout (dadKO) GSCs found that the rise time of Fused/Smurf in the preCB is higher in dadKO simulations than in wild-type [75,76,127]. Because precise control of differentiation must occur within the division time frame, the rise time of Fused is expected to be tightly regulated. In dadKO, the concentration profile takes significantly longer to respond and reach a steady state, which is in agreement with delayed differentiation observed in Dad mutants [21]. Furthermore, it has been hypothesized that Dad confers robustness to GSC signaling by enabling adaptation to environmental and intrinsic variability, both across germaria and among GSCs within the same germarium. Fused/Smurf makes the system robust by ensuring that the CBs have a low value of pMad. In dadKO ovaries, the pMad levels in GSCs would be higher, which has been hypothesized to make the pMad levels in CB higher. This loss of noise attenuation is consistent with the delayed differentiation phenotype and increased GSC number [21,78]. Similarly, a hypomorphic Dullard allele and a Dullard RNAi line exhibit similar levels of pMad throughout the G1/S phase as opposed to an asymmetric distribution of pMad in the wild-type tissues [27]. This highlights the effect of the phosphatase, Dullard, in conferring robustness in GSCs.

BMP signaling in the early *Drosophila* embryo also prioritizes rapid activation. In a 2–3-h old *Drosophila* embryo, BMP signaling patterns the dorsal half of the embryo [41,157]. Initial Smad signaling is broad and weak on the dorsal 25%–30% of the embryo. About 30 min later, the Smad signal intensifies and sharpens to a narrow domain in the dorsal-most 5%–10% of the embryo, which illustrates the rapid response time required in the embryonic *Drosophila* Smad network [7,16,158,159].

Later in *Drosophila* development, BMP controls robust and highly precise cell differentiation during vein differentiation in the pupal wing. Yan et al. (2009) demonstrated that synchronized activation of BMP and EGFR signaling arises from a bistable positive feedback loop driven by mutual activation of ligand production [103]. The bistable regime requires that both BMP and EGFR signaling act on similar timescales of tens of minutes. Furthermore, single pulses of constitutively active Type I BMP receptor Thickveins resulted in stimulation of both BMP and EGFR signaling activity, but prolonged ectopic stimulation decreased ectopic vein formation. This suggests the presence of a negative feedback mechanism operating on a longer, hour-length timescale limits the spatial spread of vein differentiation. Precise free vein patterning, therefore, requires fine-tuning of the BMP response time, which is fast enough to engage positive feedback for vein induction yet sufficiently delayed to allow longer-term negative feedback to suppress extraneous and noisy differentiation.

In zebrafish, the dorsoventral axis is patterned during embryogenesis, more specifically, late blastula and early gastrula stages, of the embryo within 3.5–8 h post fertilization (HPF) [160–162]. The BMP signaling gradient forms with remarkable speed, establishing a consistent nuclear-pSmad profile that can be immunostained, visualized, and quantified as the overall output of the signaling network. This rapid and reproducible gradient formation reflects a system optimized for fast response and temporal precision. Quantitative analyses across mutant lines have revealed how such fast patterning is coupled with developmental robustness, as the network maintains consistent outputs despite perturbations [35]. Another common method of assessing BMP activity is to categorize the 24 HPF embryo phenotype. BMP drives ventral tissue differentiation, and phenotypes have been categorized with overactive BMP lines as V1 to V4, with increasing ventralization, and lines with diminished BMP activity as C1 to C5, being dorsalized [163,164]. At the same time, the signaling gradient develops rapidly across multiple layers of signaling. The mechanisms of this cascade that provide robustness and noise attenuation have proven difficult to untangle. Greenfeld and Mullins (2021) found that BMP gene activation relied more heavily on threshold levels rather than a strict gradient shape or exposure duration [13]. Larson et. al. (2025) further suggest that noise attenuation arises from the BMP heterodimer-receptor interactions and low-pass filtering behavior downstream, uncovered with stochastic signaling [151]. Consistent with these findings, Shaikh et al. (2024) found that trade-offs in performance objectives between systems highlighted response time in rapidly developing systems such as the zebrafish embryo to be especially related to downstream phosphatase levels (Figure 5) [76].

## Discussion and open questions

*In Drosophila*, zebrafish, and hPSCs, there is a remarkable conservation of core components of the SMAD pathway, contrasted with a striking diversity in regulatory strategies. By comparing the roles of BMP and its orthologs, we can understand how adaptations in this fundamental signaling network regulate cell differentiation through comparison of the functional roles of BMP and its orthologs in diverse systems. For instance, in *Drosophila* embryos BMP gradient formation depends on a ‘shuttling’ mechanism involving Sog and Tld [23], while the wing disc primarily relies on restricted extracellular diffusion to spread Dpp [49]. Alternatively, zebrafish establish their BMP gradient via a ‘source-sink’ mechanism and feedback loops, which cross-talk with other signaling pathways, and extracellular modulators help stabilize and refine the gradient [79,84]. Moreover, the interpretation of this signal is not only determined by spatial gradients but is strongly influenced by signaling dynamics that are precisely tailored to the biological context. For instance, the *Drosophila* embryo decodes through a dynamic and temporally integrated response to determine downstream gene expression, rather than instantaneous concentration alone. This dynamic decoding activates a tiered (Type I–III) gene response to specify multiple cell fates across the dorsoventral axis [16,43,114,115,165], with receptor-ligand binding kinetics and feedback regulation contributing to the robustness of BMP signaling [40,46]. In contrast, the GSC niche uses the pathway as a switch: high BMP signaling represses the differentiation gene *bam* to maintain stemness, a steep transition that is spatially defined by asymmetric division and sharpened by negative feedback from the inhibitory Smad, *Dad*.

These diverse interpretive strategies all showcase the use of complex feedback networks to ensure the robustness of developmental patterning, while simultaneously having functional behavior that depends on cellular and tissue-level context. This context-dependency means each system must balance competing POs [76]. For instance, the *Drosophila* GSC niche optimizes for noise filtering to create a sharp, binary ‘off’ signal, potentially at the cost of a slower response. In contrast, the early *Drosophila* and zebrafish embryos prioritize rapid response times to meet tight developmental schedules, achieving stability and precision through means such as threshold-based interpretation and extracellular motifs [7,133,153,154,155,156,158,159,166]. Identifying how these trade-offs are balanced is a key knowledge gap. However, attempts to quantitatively formalize the trade-offs among multiple competing objectives across model systems and specifically manipulate these competing objectives across these systems are still lacking, which hinders the discovery of generalized biological rules [167]. Bridging this gap will demand precise, quantitative access to the Smad pathway’s inputs and outputs, which is essential for measuring key network response dynamics (e.g., response time and noise attenuation) and for mapping the quantitative relationship between BMP signaling and nuclear pSmad activity and other underlying biophysical interactions.

The BMP/Smad pathway is an ideal system to pioneer this quantitative, cross-species approach because of its high conservation across species. Modeling this network has already proven invaluable for describing gradient formation, resolving conflicting hypotheses like long-range Dpp transport, and understanding how complex, emergent properties arise. The pathway’s power as a model system arises from a central dichotomy: its core topology and protein functions are highly conserved, while the protein concentrations and the parameters they control, like dephosphorylation and nuclear transport rates, are not conserved and operate in a highly context-dependent manner [2,168–170]. This presents an opportunity to leverage the system’s conserved architecture to build a predictive, cross-species computational model to overcome the limitations of siloed, ad hoc studies [171]. By integrating experimental data from *Drosophila*, zebrafish, and hPSCs, a cross-species model could predict differences in the balance of PO trade-offs and identify the most influential non-conserved parameters. Validation of this model, along with a unified toolkit of characterized BMP tools, would allow discoveries in one system to immediately and directly inform our knowledge of others, enabling a more holistic understanding of the regulatory features that drive context-specific cell behavior.

Computational models of the BMP-Smad signaling pathway offer powerful tools for cross-species optimization and translational prediction. As quantitative datasets accumulate across *Drosophila*, zebrafish, and human pluripotent stem cell systems, it is becoming feasible to build multi-objective modeling frameworks that identify conserved design principles while capturing species-specific constraints. The ‘Digital Cousins’ framework demonstrates this concept by using multi-objective optimization to fit a single conserved BMP model to both *Drosophila* and zebrafish datasets, allowing shared pathway topology to be compared with species-specific parameter variation [152]. Thus, cross-species digital cousins provide a systematic way to distinguish conserved pathway architecture from context-dependent regulatory mechanisms, improving the interpretability and translational relevance of developmental signaling models. These models also create a path toward computational drug screening that leverages variation in signaling pathways and mechanisms to evaluate pathway perturbations across multiple biological backgrounds before moving into human systems, especially in BMP pathway-targeted cancer treatment. Emerging AI and machine-learning approaches—including physics-informed neural networks, generative models of signaling dynamics, and AI-accelerated parameter inference—are transforming the scale at which these models can be trained, optimized, and validated and could be tightly integrated with existing mechanistic models to provide a predictive, cross-species, and clinically actionable model.

## Open Access

Open access for this article was enabled by the participation of Purdue University in an all-inclusive Read & Publish agreement with Portland Press and the Biochemical Society under an agreement with Big Ten Alliance.

## Abbreviations

ACV: anterior crossvein
AECs: anterior escort cells
APF: after pupal formation
ID: inhibitor of differentiation
BAMBI: BMP and activin membrane-bound inhibitor
BMP: Bone-Morphogenetic Protein
CBs: cystoblasts
Co-SMADs: common partner SMADs
CpCs: cap cells
CV-2: crossveinless-2
CVs: crossveins
CTDNEP1: C-terminal domain nuclear envelope phosphatase 1
Dad: daughters against Dpp
dadKO: *dad* knockout
Dpp: decapentaplegic
ECM: extracellular matrix
EGFR: epidermal growth factor receptor
Fu: Fused
GSCs: germline stem cells
GRN: gene regulatory networks
GPI: glycosylphosphatidylinositol
HSPGs: heparan sulfate proteoglycans
HPF: hours post fertilization
hPSCs: human pluripotent stem cells
I-Smads: inhibitory Smads
LVs: longitudinal veins
MAPK: mitogen-activated protein kinase
NCPs: non-conserved parameters
PDEs: partial differential equations
Pent: Pentagone
POs: performance objectives
PCV: posterior crossvein
PPases: phosphatases
pSMAD/pMad: phosphorylated Smad
RMT: receptor-mediated transcytosis
R-SMAD: receptor-regulated SMAD
Sog: short gastrulation
TGF-β: transforming growth factor β
Tld: Tolloid
Tlr: Tolloid-related
Tkv: thickveins
Tsg: twisted gastrulation
Vg: Vestigial
Vkg: Viking
